# Metagenomic Insights Into a Cellulose-Rich Niche Reveal Microbial Cooperation in Cellulose Degradation

**DOI:** 10.3389/fmicb.2019.00618

**Published:** 2019-03-28

**Authors:** Jinming Cui, Guoqin Mai, Zuowei Wang, Quan Liu, Yan Zhou, Yingfei Ma, Chenli Liu

**Affiliations:** ^1^Institute of Synthetic Biology – Shenzhen Institutes of Advanced Technology, Chinese Academy of Sciences, Shenzhen, China; ^2^Guangzhou Institute of Advanced Technology, Chinese Academy of Sciences, Guangzhou, China; ^3^Shenzhen Institute of Synthetic Biology, Shenzhen Institutes of Advanced Technology, Chinese Academy of Sciences, Shenzhen, China

**Keywords:** metagenomics, mutualistic interaction, cellulolytic process, cellulose-degrading microbial community, genomic sequencing

## Abstract

**Background:**

Cellulose is the most abundant organic polymer mainly produced by plants in nature. It is insoluble and highly resistant to enzymatic hydrolysis. Cellulolytic microorganisms that are capable of producing a battery of related enzymes play an important role in recycling cellulose-rich plant biomass. Effective cellulose degradation by multiple synergic microorganisms has been observed within a defined microbial consortium in the lab culture. Metagenomic analysis may enable us to understand how microbes cooperate in cellulose degradation in a more complex microbial free-living ecosystem in nature.

**Results:**

Here we investigated a typical cellulose-rich and alkaline niche where constituent microbes survive through inter-genera cooperation in cellulose utilization. The niche has been generated in an ancient paper-making plant, which has served as an isolated habitat for over 7 centuries. Combined amplicon-based sequencing of 16S rRNA genes and metagenomic sequencing, our analyses showed a microbial composition with 6 dominant genera including *Cloacibacterium, Paludibacter, Exiguobacterium, Acetivibrio, Tolumonas*, and *Clostridium* in this cellulose-rich niche; the composition is distinct from other cellulose-rich niches including a modern paper mill, bamboo soil, wild giant panda guts, and termite hindguts. In total, 11,676 genes of 96 glucoside hydrolase (GH) families, as well as 1,744 genes of carbohydrate transporters were identified, and modeling analysis of two representative genes suggested that these glucoside hydrolases likely evolved to adapt to alkaline environments. Further reconstruction of the microbial draft genomes by binning the assembled contigs predicted a mutualistic interaction between the dominant microbes regarding the cellulolytic process in the niche, with *Paludibacter* and *Clostridium* acting as helpers that produce endoglucanases, and *Cloacibacterium, Exiguobacterium, Acetivibrio*, and *Tolumonas* being beneficiaries that cross-feed on the cellodextrins by oligosaccharide uptake.

**Conclusion:**

The analysis of the key genes involved in cellulose degradation and reconstruction of the microbial draft genomes by binning the assembled contigs predicted a mutualistic interaction based on public goods regarding the cellulolytic process in the niche, suggesting that in the studied microbial consortium, free-living bacteria likely survive on each other by acquisition and exchange of metabolites. Knowledge gained from this study will facilitate the design of complex microbial communities with a better performance in industrial bioprocesses.

## Introduction

Cellulose, the main component of the cell wall of green plants, algae, and oomycetes, is the most abundant organic polymer on Earth, and has an undeniably central role in the global carbon cycle (Klemm et al., 2005). Consequently, cellulolytic processes occur widely in the Earth’s biosphere. In these processes, microbes employ cellulases to break down cellulose into oligosaccharides called cellodextrins or completely into glucose units, followed by sugar uptake and assimilation.

With the cellulolytic process providing a carbon and energy source, microbes proliferate and many microbial ecosystems evolve. For example, symbiotic microorganisms in the guts help some animals, particularly ruminants, giant pandas, and termites, digest cellulose; also, microbial consortia constantly recycle the cellulose-rich plant biomass in soil environments. A few examples of these ecosystems have been investigated to understand their function and dynamics (Warnecke et al., 2007; Zhu et al., 2011; He et al., 2013; Zhou et al., 2014; Lin et al., 2015).

In addition to natural recycling, cellulose has historically been used to produce paper. A fascinating example lies in an ancient village named Dengcun in Guangdong Province, China, where people have been using local bamboo and water resources to make paper continuously for 7 centuries. The centuries-long papermaking process has generated a special cellulose-rich and alkaline niche, and presumably a particular microbial ecosystem has been evolving *in situ*. Two intriguing questions can be raised: (i) what is the composition of the microbial ecosystem; (ii) what roles are these microbes playing in the cellulolytic process?

Cellulose is a sustainable carbon and energy source, and thus cellulolytic microbes and their enzymes can play important roles in industrial applications, such as biofuel production (Carroll and Somerville, 2009). Many studies have focused on single cellulolytic species based on a culture-dependent method, such as *Clostridium thermocellum, C. cellulolyticum*, and *Caldicellulosiruptor bescii*, most of which belong to the phylum Firmicutes (Demain et al., 2005; Dash et al., 2016). Nevertheless, the natural cellulolytic process is usually interplay within a microbial consortium. According to the BQH, in a given ecosystem, the evolution of the microbial community tends to make the constituent microbes rely on each other (Morris et al., 2012). Microbial cells (or species) can be considered as players competing and cooperating *in situ*, and they likely would have employed an ESS and become highly efficient in, for example, cellulose degradation (Hummert et al., 2014). In this study, we aimed to reveal the microbial composition and adaption mechanisms of the special cellulolytic ecosystem in Dengcun. Metagenomic data were analyzed and compared with the results from other samples rich in bamboo, i.e., bamboo soil (Lin et al., 2015) and wild giant panda guts (Zhu et al., 2011). In addition, modern paper mill (Lu and Lu, 2014) and termite hindguts (He et al., 2013) samples were also included for comparison. New cellulolytic species, as well as the ESS revealed by the culture-independent metagenomic analysis method in this study, can improve the future design of synthetic microbial consortia for cellulose conversion (Kudo et al., 1987; Kato et al., 2004, 2005; Salimi et al., 2010).

## Materials and Methods

### Sample Collection

Dengcun is located at longitude 112.588° E, latitude 23.3268° N, and at an altitude of 67.5 m. APMP samples 1 and 2 were collected from a bamboo fiber soaking pit (water depth 30 cm), and a bamboo pulp pit (water depth 120 cm), respectively, of an ancient paper-making plant in the biggest local workshop. After stirring, 1.5 L of liquid/pulp mixtures of each sample were collected in centrifuge bottles and shipped at 4°C, then frozen and stored at -80°C. Water quality and parameters are listed in Supplementary Table S1. The concentrations of total phosphorus (TP), total nitrogen (TN), dissolved organic carbon (DOC), ammonium (NH4-N), nitrate (NO3-N), nitrite (NO2-N), and soluble phosphorus (PO4-P) were measured using standard methods (Gilcreas, 1967).

To extract bacterial DNA, samples were mixed using a vortex for 5 min, followed by centrifugation at 100 g for 10 min to separate the bamboo fibers. Fifty mL of the supernatants were spun down at 4,600 g for 30 min, and the precipitates were washed twice with 10 mM pH 7.4 potassium phosphate buffer. In both samples, the pellets appeared blackish, and pea-sized samples were taken and the bacterial genomic DNA was extracted using the Rapid Soil DNA Isolation Kit by Sangon Biotech Co., Ltd., (Shanghai). Clear bands of genomic DNA were observed using polyacrylamide gel electrophoresis.

### 16S rRNA Gene Amplicon-Based Sequencing

Briefly, primer pairs targeting the 16S rRNA gene v3-v4 region (515–806) were employed for amplicon-based sequencing on total DNA of samples 1 and 2, respectively. The F515 primer (5′TATGGTAATTGTGTGCCAGCMGCCGCGGTA A3′) was used across all samples. We added a linker and two barcode sequences at the 5′ end of R803 primer (5′AGTCAGTCAGCCGGACTACHVGGGTWTCTAAT3′), for sequencing of pooled samples (Supplementary Table S2). PCRs were set up containing 2 μl of APMP genomic DNA as template, 2 μl of F515 primer, 2 μl of R803 primer, 4 μl of dNTP, 4 μl of 25 mmol l^-1^ MgCl2, 5 μl of 10 × Ex Taq buffer, 0.25 μl Taq polymerase (5 U μl^-1^), and 30.75 μl of distilled deionized water. PCR began with 98°C 1 min, followed by 30 cycles of 98°C 10 s, 58°C 30 s, 72°C 2 min, and finally 72°C 10 min. The PCR products were purified and preserved in 25 μl sterile water using the San-Prep column DNA gel extraction kit (Sangon Biotech, Shanhai, China). Concentrations of the purified PCR products were determined using NanoDrop (Thermo fisher Scientific Inc. Wltham, MA, United States); 200 ng of the purified PCR products of each sample were pooled together equally and then were sent to Novogene Genomics Co., Ltd., (Beijing) for Illumina Mi-Seq PE250 platform. The generated reads were firstly filtered by quality (Q > 30), and the high quality paired-end reads were assembled into a complete sequence with a minimum overlap of 50 bp using the software Qiime (Caporaso et al., 2010). Sequences shorter than 300 bp with an expected error of more than 0.5 were discarded. Chimera checking of the sequences was performed using the software Qiime. Sequences were assigned to samples according to the barcodes. Barcodes and primers were removed from the sequences for further analysis. The sequencing data have been deposited in NCBI GenBank with the accession numbers SAMN08818942.

### Taxonomic Classification of 16S rRNA Gene Sequences

16S rRNA gene amplicon-based sequencing reads were assigned to OTUs at genus and phylum levels according to the method described by Ravel et al. (2011). Briefly, the RDPII classifier was employed (Wang et al., 2007): if the median RDP confidence value of a 16S rRNA gene sequence assigned to a genus was less than the 0.5 thresholds, the median RDP score for the next-higher taxonomic level (family) was considered. If it was above the RDP confidence value threshold (0.5) then the OTU was named as “FamilyName_genus-name.” For each taxonomic level, the algorithm was applied in an iterative manner until the RDP confidence value was above 0.5. A shell script was employed to count the number of sequences for each genus in each sample. Genera that only appeared in one sample were discarded. PCA was performed by the software of PAST (version 3.20) based on the relative abundance (Hammer, 2001).

Published sequencing data of the samples from a modern paper mill (Lu and Lu, 2014), bamboo soil (Lin et al., 2015), wild giant panda guts (Zhu et al., 2011), and termite hindguts (He et al., 2013) are listed in Supplementary Table S3 and were also analyzed in this study using the same bioinformatic pipelines.

### Metagenomic Sequencing, *de novo* Assembly, Gene Prediction, and Annotation

Sixty μL of 300–500 ng/μL DNA of samples 1 and 2 were submitted for metagenomic sequencing to Berry Genomics Co., Ltd., (Beijing). Whole metagenomic shotgun sequencing was performed using the Illumina Hi-Seq2500 V4 PE125 (Paired-end 125 bp) platform. Briefly, whole bacterial genomic DNA was treated using a next-generation sequencing Fast DNA Library Prep Set for Illumina Hi-Seq 2500 V4 PE125 (Paired-end 125 bp) to prepare the metagenomic library following a standard Illumina protocol. The retrieved DNA library was evaluated by the StepOnePlus Real-Time PCR system (Applied Biosystems), and considered to be qualified showing a single peak with a concentration above 3 nM and a volume above 25 μL. The generated sequencing reads were firstly filtered by quality. Low-quality reads (reads containing adaptor sequence, reads with *N* > 3%, reads where ≥50% of the bases had a Q score ≤30) were removed. Clean reads were assembled by MetaVelvet (Namiki et al., 2012) with the default parameter of k-mer size of 39. Contigs larger than 300 bp were kept for further analysis. Statistics of the sequencing and assembly data were listed in Supplementary Table S4, and the data have been deposited in NCBI GenBank with the accession numbers SAMN07807411 for sample 1 and SAMN07807412 for sample 2.

Genes encoded by the contigs (≥300 bp) were predicted by MetaGeneMark (Tang et al., 2013). The clusters of orthologous groups (COG) database was downloaded from NCBI and was formatted as local COG database^1^ using the program Makeblastdb (Galperin et al., 2015). All protein sequences derived from the NCBI bacterial genomes were formatted as a local database (Latest Bacterial protein sequence database, LBPSDB) using the program Makeblastdb^2^. The predicted genes were further annotated functionally by the program Blastp against the COG database and the LBPSDB (Galperin et al., 2015) with cutoff e-value 1e-10. The taxonomic affiliation of each gene was assigned at the genus level according to the best-hit counterpart in the LBPSDB. The number of the APMP predicted proteins of each genus was counted, and the mean of identities and the standard deviation (SD) between the APMP protein sequences and their counterparts in the LBPSDB database were calculated, respectively (Supplementary Table S5). In addition, the predicted protein sequences corresponding to the top abundant genera in APMP samples were extracted, respectively and then were uploaded to KEGG mapper^3^ (Kanehisa et al., 2016), to identify cellulose-degrading enzymes and cellodextrin transporters in the reconstructed KEGG pathways (Supplementary Table S5).

### Cellulase Analysis

With reference to the method of this article (Warnecke et al., 2007), the predicted protein sequences encoded by the contigs (≥300 bp) were compared against the dbCAN database^4^ by HMMER 3.0 hmmscan with cutoff evalue 1e-10 (Warnecke et al., 2007). The dbCAN database is a knowledge-based resource specialized in the enzymes that bind, synthesize, or break down complex carbohydrates (Lombard et al., 2014a). The predicted proteins with functional domains of GH and carbohydrate binding modules (CBM) were assigned as putative cellulase for further analysis. GHs and CBMs were named according to the carbohydrate-active enzyme (CAZy) nomenclature scheme (Cantarel et al., 2009; Lombard et al., 2014b). The genes of 13 GH families that encode cellulases, namely GH5-9, GH12, GH44, GH45, GH48, GH51, GH61, GH74, and GH124 (Sukharnikov et al., 2011), were further verified by being compared with homologs in the non-redundant protein database of Genbank by BLASTP. The number of the APMP genes in each GH family was given in Supplementary Table S6.

All full-length genes of the 13 GH families were listed in Supplementary Table S7, and functional domains, including GH and CBM, were also recognized with the software HMMER 3.0 hmmscan. Gene-40972 of the GH5 family and gene-6561 of the GH9 family were taken as examples, due to their high homologies with their counterparts in the RCSB PDB database^5^. The amino acid compositions, charges, and isoelectric points of gene-40972 and gene-6561, together with their homologs, were analyzed by EMBOSS Pepstats (Li et al., 2015). The protein structures of gene-40972 and gene-6561 were predicted using the Galaxy template-based modeling program (Shin et al., 2014). *C. thermocellum* CtCelC (pdb ID: 1ceo) was used as a template for gene-40972 (Dominguez et al., 1996); *Alicyclobacillus acidocaldarius* AaCel9A (pdb ID: 3gzk) and leaf-branch compost LC-CelG (pdb ID: 3x17) were used as templates for gene-6561 (Eckert et al., 2009; Okano et al., 2015). The ligand cellotriose was predicted and docked into the models by GalaxySite (Shin et al., 2014). All structure figures were prepared using the PyMOL molecular graphics system.

### Carbohydrate Transporters Analysis

All genes predicted from the APMP metagenomic contigs (≥300 bp) were compared against the TCDB^6^ by the program HMMER 3.0 hmmscan with an E-value 1e-10 to identify the genes encoding carbohydrate transporters. The genes with hits were counted and the corresponding protein sequences were selected as carbohydrate transporters for further analyses. The figures were drawn by IBS (Liu et al., 2015).

### Binning Metagenomic Contigs by Vizbin

Reference-independent approaches were sought to cluster the metagenomic contigs (binning) by exploiting the inherent genomic signatures. In order to get high coverage of the bacterial genomes, the contigs of two APMP samples were combined for binning analysis and the larger contigs (≥1000 bp) were clustered by Vizbin (Laczny et al., 2015)with the default parameters: Minimal contig length 1000, Number of threads 1, Kmer length 5, Intermediate dimensions 50, Theta 0.5, Perplexity 30.0, seed 0. The coding genes of these contigs were analyzed by AmphoraNet for searching phylogenetic marker genes to estimate the taxonomic composition of the bacterial community of the metagenomes^7^ (Supplementary Table S8; Kerepesi et al., 2014). The contigs carrying the marker genes of the top abundant genera were marked using different colored stars to show the taxonomy of each cluster where the corresponding marker genes exist. Meanwhile, the contigs carrying the genes encoding endoglucanase, beta glucosidase, 6-phospho-beta-glucosidase, and cellobiose phosphorylase, were marked using different patterned stars to show the function of the phylotype represented by each cluster.

## Results

### Profiling of the Microbial Consortium in the Ancient Paper-Making Pulp (APMP) Samples

**FIGURE 1:**
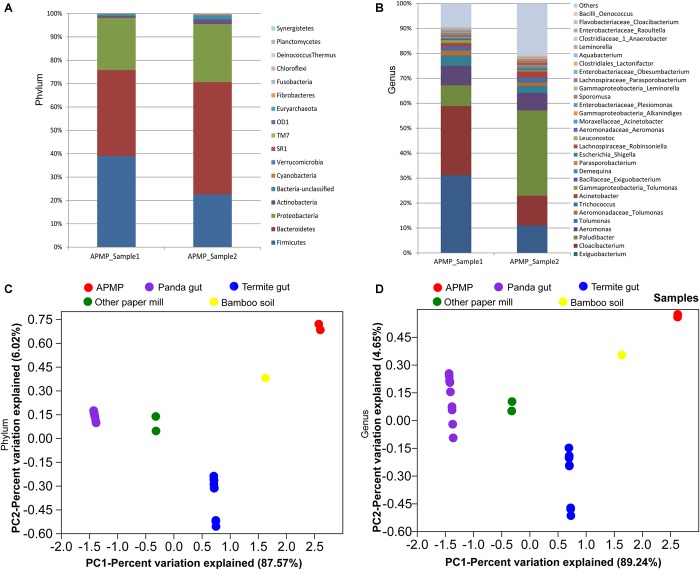
Microbial compositions in APMP samples. Relative abundances at the phylum level **(A)** and the genus level **(B)** based on the 16S rRNA gene amplicon-based sequencing. Comparison of the microbial communities in various environments at the phylum level **(C)** and genus level **(D)** by PCA analysis.

We profiled the microbial consortium inhabiting the APMP ecosystem by analysis of the 16S rRNA genes. In total, 15470 raw reads were obtained. After quality filter and assembly of the paired-end reads, 2581 and 3486 sequences were generated in APMP samples 1 and 2, respectively (Supplementary Figure S1). In total, 8 and 17 phyla, and 207 and 331 genera were identified in APMP samples 1 and 2, respectively. On average, 98.21% of the total reads were assigned to 4 phyla, namely Bacteroidetes (42.44%), Firmicutes (30.79%), Proteobacteria (23.58%), and Actinobacteria (1.40%) in both APMP samples (Figure 1A). The most abundant genera (in samples 1 and 2, respectively) were *Cloacibacterium* (27.82/11.91%), *Paludibacter* (8.29/34.20%), *Exiguobacterium* (31.08/11.01%), *Tolumonas* (4.18/2.79 %), and *Aeromonas* (7.81/27.02%) (Figure 1B). Members of the five genera are likely obligate or facultative anaerobes.

The microbial compositions were distinct among these samples, with the most abundant phylum being Bacteroidetes (33.43%) in the modern paper mill sample (Lu and Lu, 2014); Acidobacteria (37.76%) in the bamboo-soil sample (Lin et al., 2015); Firmicutes (56.97%) in the bamboo-feeding wild giant panda gut sample (Zhu et al., 2011); and Spirochaetes (56.61%) in the wood-feeding termite hindgut sample (He et al., 2013). PCA analysis of the genus distribution data indicated that the microbial communities of APMP samples 1 and 2 were similar to each other and somewhat close to the bamboo soil sample, but different from other samples (Figure 1C,D and Supplementary Tables S9,S10).

### Profiling of GH Families and Cellulases in the APMP Consortium

We next used a metagenomic approach based on next-generation *de novo* sequencing to identify the functional attributes encoded in the APMP consortium, with a focus on the cellulolytic microbes and their functional genes. The metagenomic sequencing generated 20 million and 23 million high-quality reads in samples 1 and 2, respectively. Assembly of the reads resulted in 172,009 and 186,534 total contigs, respectively (≥300 bp); among them, 23,086 contigs in sample 1 and 25,201 contigs in sample 2 are larger than 1000 bp. The largest contig is 122,944 bp in length. In total, 223,510 and 249,163 coding genes were predicted in samples 1 and 2, respectively; 55.56% of the genes in sample 1 and 55.48% of the genes in sample 2 were assigned putative functions based on the COG database. Sequencing and assembly statistics are given in Supplementary Table S4.

**FIGURE 2:**
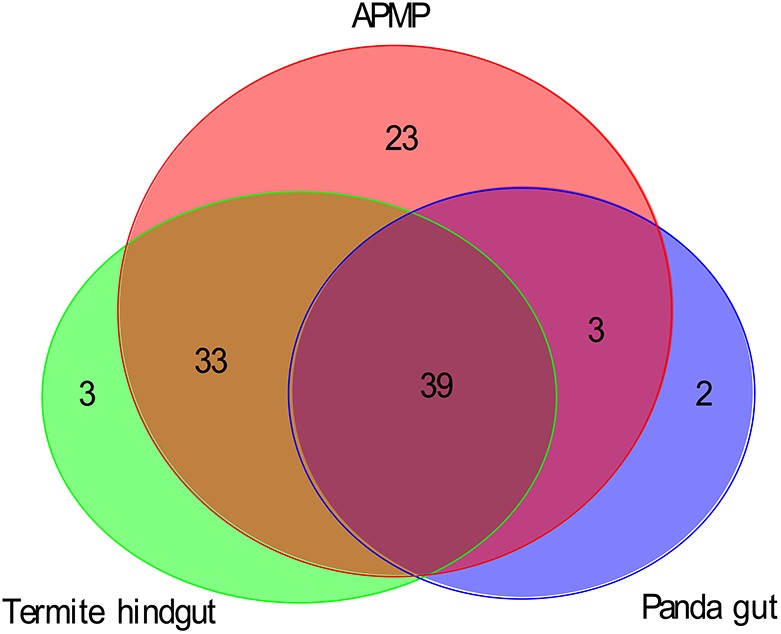
Venn diagram of the GH families in the samples of APMP, panda gut, and termite hindgut.

Using the dbCAN database as a reference, we assessed the functional capacities of the APMP samples and identified 5808 genes of 92 GH families in APMP sample 1, and 5868 genes of 93 GH families in APMP sample 2. In total, 98 GH families were present in APMP samples, and 87 GH families were present in both samples. By contrast, only 448 genes of 44 GH families were identified in the giant panda gut samples (Zhu et al., 2011), and 2779 genes of 75 GH families were identified in the termite hindgut sample (Warnecke et al., 2007; Supplementary Table S6). The distribution of the GH families among the three types of samples is summarized in Figure 2. Thirty-nine GH families were present in all three samples. The majority of the identified GH families in the panda gut (42/44) and termite hindgut (72/75), respectively, were shared with the APMP samples. Twenty-three GH families were present in the APMP samples only (Supplementary Table S6).

**FIGURE 3:**
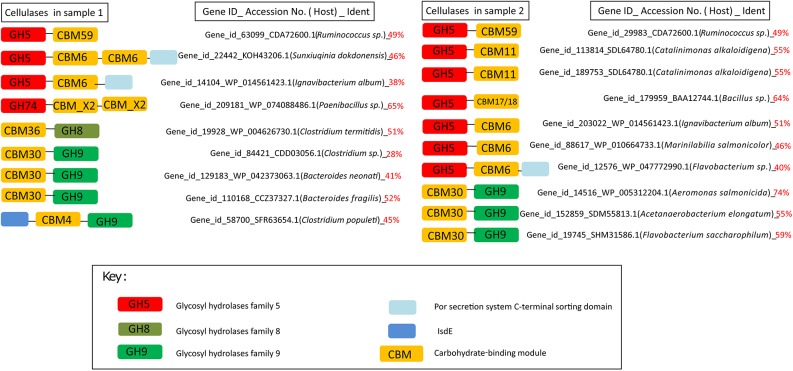
Representative domain architectures of the cellulases. The functional domains were detected on the identified cellulases of the APMP samples using HMMER 3.0 hmmscan search against the dbCAN database. The accession numbers, host bacterial species and percent identities of the best hits of each cellulase in the NCBI-nr database were shown.

Cellulases, a heterogeneous group of enzymes that mediate cellulose degradation, have been found within 13 GH families (GH 5–9, 12, 44, 45, 48, 51, 61, 74, and 124) (Sukharnikov et al., 2011; Okano et al., 2015). Notably, these families are unrelated and diverse in sequence, and their domain linkage between the GH domains and the CBM domains had been proposed (Sukharnikov et al., 2011). The architectures of the cellulase genes were analyzed and representative results were shown in Figure 3. Seven distinct CBM types (CBM 6, X2, 36, 11, 17/18, 30) were located on these genes. Notably, CBM30 was frequently linked to GH9. These cellulases have low identities (28–74%) with their counterparts from various bacterial phylotypes in the NCBI-nr database, suggesting that the APMP cellulase genes originated from divergent bacterial hosts.

All full-length genes belonging to the 13 GH families in samples 1 and 2 are listed in Supplementary Table S7 with their homologous templates and putative functions. Two representatives, gene-40972 (GH5) and gene-6561 (GH9) showed high homologies with their homologs in the RCSB PDB database, therefore they were chosen as examples for analysis using the Galaxy template-based modeling program (Shin et al., 2014).

**FIGURE 4:**
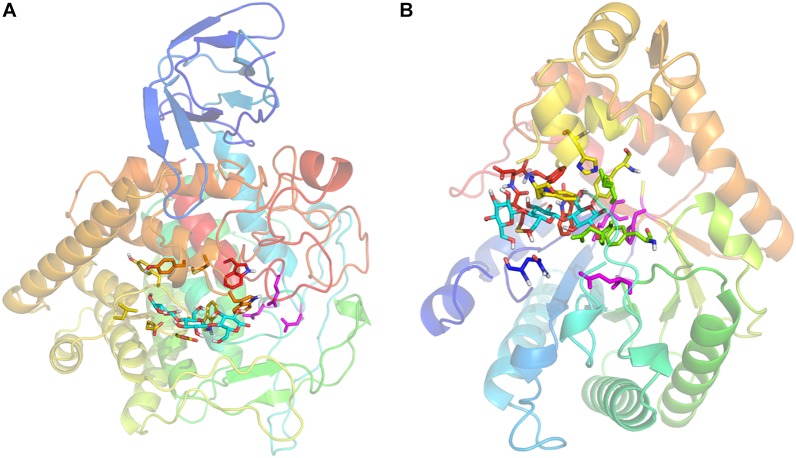
Homology models of gene-6561 **(A)** and gene-40972 **(B)** from the APMP samples. The protein backbone is shown as a transparent cartoon, and colored with a rainbow spectrum (N-terminal blue, C-terminal red). **(A)** A cellotriose molecule (cyan sticks) is modeled by GalaxySite, occupying the –1, –2, and –3 subsites. Interacting residues in the binding pocket are shown as sticks, including L342, G344, Y345, G346, W347, E348, E349 (yellow), W405, M409, Y410 (orange), and W521 (red). Catalytic residues D149, D152, and E516 of the active site are shown as magenta sticks. **(B)** A cellotriose molecule (cyan sticks) is modeled by GalaxySite, occupying the –1, –2, and –3 subsites. Interacting residues in the binding pocket are shown as sticks, including Q16 (blue), D100, T102, Y182 (lime green), Y206, H213, W218 (yellow), W320, M325, D326, and F327 (red). Catalytic residues E146, H204, and E287 of the active site are shown as magenta sticks.

The cellulase encoded by gene-40972 was predicted to fold into a cylindrical (α/β)_8_-barrel with a catalytic pocket on the C-terminal side of the barrel, typical of GH5 cellulases. The cellulase encoded by gene-6561 was predicted to contain a catalytic domain with a GH9 signature (α/α)_6_-barrel fold, and an N-terminal immunoglobulin-like (Ig-like) domain. The substrate binding pockets and the catalytic residues of gene-40972 (Figure 4A) and gene-6561 (Figure 4B) were highly similar to their structural homologs, *C. thermocellum* CtCelC and LC-CelG, respectively (Dominguez et al., 1995; Kerepesi et al., 2014; Laczny et al., 2015; Liu et al., 2015; Okano et al., 2015). In both cases, a cellotriose molecule was predicted to bind to the substrate pocket, and the catalytic residues D149, D152, and E516 of gene-6561, and E146, H204, and E287 of gene-40972 were all conserved. The homologous structures suggest they have conserved the cellulase functions.

Analysis of amino acid compositions, charges, and isoelectric points of the two genes and their homologs indicates that the cellulase of gene-40972 has similar properties to alkaline cellulase 103 from *Bacillus sp*. found in soda lakes, e.g., isoelectric points closer to 4 and negative charges lower than -20, but was different from CtCelC and the FnCel5A (Supplementary Table S11; Dominguez et al., 1995; Shaw et al., 2002; Zheng et al., 2012). Notably, both gene-40972 and 103 cellulases have a marked decrease in Lys residues, as was suggested to be an alkaline adaptation mechanism for the industrial cellulase K (Shirai et al., 2001). The cellulase of gene-6561 was also predicted to have an isoelectric point close to 4 and a negative charge lower than -20 (Supplementary Table S11). However, when compared to its structural homologs, LC-CelG, AaCel9A, and CtCelD (Chauvaux et al., 1995; Dominguez et al., 1995; Shirai et al., 2001; Shaw et al., 2002; Eckert et al., 2009; Zheng et al., 2012; Kerepesi et al., 2014; Laczny et al., 2015; Liu et al., 2015; Okano et al., 2015), gene-6561 cellulase unexpectedly has an increased number of Lys residues, and strikingly doubled the amount of Glu residues, resulting in its low isoelectric point. It is likely gene-6561 cellulase of the GH9 family employs a different alkaline adaptation mechanism from the GH5 family cellulases.

### The Classification of “Beneficiaries” and “Helpers” Regarding Cellulose Degradation in the APMP Consortium

In the APMP consortium where cellulose is the dominant carbon source, microbes produce cellulases to sustain their growth. We also investigated the gene content of the metagenomes and assigned the genes taxonomic origins according to the best hits in the LBPSDB. The genera of *Cloacibacterium, Paludibacter, Exiguobacterium, Acetivibrio, Tolumonas*, and *Clostridium* have the highest numbers of protein sequences in the APMP samples (Supplementary Table S5). Most (95.1%) of the protein sequences encoded by the genera showed less than 90% identities with their counterparts in the LBPSDB, indicating that the APMP strains belong to novel species of the genera.

Further, the protein sequences assigned to the genera were predicted in KEGG and the proteins involved in cellulose-degrading pathways were determined. In total, 1744 carbohydrate transporter genes were identified in the APMP samples (Supplementary Table S12); among them, 157 genes encode cellobiose-specific PTS family homologs of CelC, CelB, and CelA (Parker and Hall, 1990), and 85 genes encode cellobiose-specific ABC family homologs of CebE, CebF, CebG, and MsiK (Schlosser et al., 1999). Accordingly, in *Clostridium*, genes encoding endoglucanase [EC:3.2.1.4], beta-glucosidase [EC:3.2.1.21], 6-phospho-beta-glucosidase [EC:3.2.1.86], as well as PTS transporter homologs of CelC, CelB, and CelA were identified, implying that *Clostridium* species likely function as helpers and can utilize cellulose by themselves. In *Paludibacter*, genes encoding endoglucanase, beta-glucosidase, and cellobiose phosphorylase were identified, but they lacked related transporters. Note that only two bacterial cellodextrin transporter families have been characterized and reported to date (Parker and Hall, 1990; Schlosser et al., 1999); thus, it is likely that *Paludibacter* species encode an unclassified cellodextrin transporter and also function as helpers.

In *Cloacibacterium, Exiguobacterium, Acetivibrio*, and *Tolumonas*, genes encoding intracellular-process-related enzymes such as beta-glucosidases and 6-phospho-beta-glucosidases were identified, but endoglucanases were absent, suggesting that these four genera function as beneficiaries in the APMP consortium. The genes encoding cellodextrin transporter systems, such as homologs of CelC, CelB, and CelA, were also found in *Exiguobacterium, Acetivibrio*, and *Tolumonas*, but were absent in *Cloacibacterium* possibly due to low homology.

Thus, our analysis suggests that these dominant genera play distinct roles in cellulose-degrading process in APMP consortium. The genera *Clostridium and Paludibacter* can produce cellulases generating cellodextrin products that diffuse and are shared by the whole community. The other four genera survive by importing and assimilating the generated cellodextrin products. According to the BQH, the genera *Clostridium* and *Paludibacter* can be designated as “ helpers,” the other four genera as “beneficiaries” (Morris et al., 2012).

### Binning of the Metagenomic Data Supporting the Classification of Beneficiaries and Helpers

**FIGURE 5:**
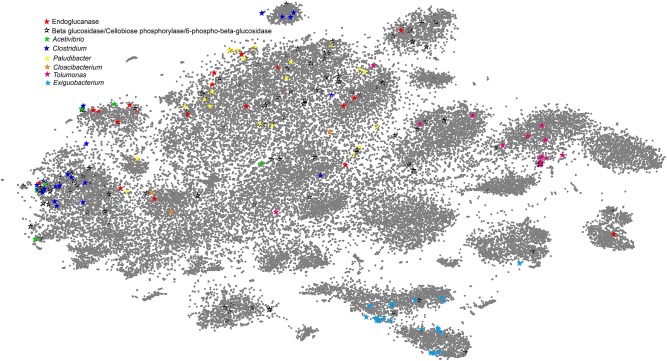
VizBin-based visualization of clusters by binning the APMP metagenomic data. The assembled contigs with lengths greater than 1,000 bp were selected for binning. The gray points represent the binned contigs. The stars represent the marker genes encoded by the contigs. Deep red stars represent the contigs encoding endoglucanases, and hollow black stars represent the contigs encoding beta glucosidases, cellobioses phosphorylases, or 6-phospho-beta-glucosidases. The remaining stars of different colors represent the contigs encoding the marker genes (Supplementary Table S8) of the six dominant genera.

Similarity-based taxonomic assignment of the genes predicted from the environmental metagenomic dataset may result in bias because of a limited number of bacteria with sequenced genomes so far. To validate the above observations, we combined the sequencing data of the two APMP samples and employed a reference-independent binning approach to cluster the assembled microbial metagenomic contigs. The contigs were labeled using the phylogenetic marker genes (Supplementary Table S8) and the genes corresponding to cellulose-degradation derived from the APMP metagenomic dataset (Figure 5).

In Figure 5, the clusters affiliated to *Clostridium, Paludibacter, Exiguobacterium*, and *Tolumonas*, respectively, can be readily recognized as we can see that the contigs with the corresponding phylogenetic marker genes intensively appear in distinct clusters. *Paludibacter* is one of the most abundant genera. In Figure 5, the marker genes of the *Paludibacter* genus lie in the largest cluster, but highly scattered distribution of the marker genes likely suggests that *Paludibacter* species are diverse in the consortium. This case also can be observed that the phylogenetic marker genes of *Cloacibacterium* and *Acetivibrio* scatter among different clusters. Significantly, most of the cellulose-degrading genes are adjacent to the marker genes of the six genera in Figure 5, suggesting that the cellulose-degrading genes are mainly carried by these top abundant genera, the main players in the APMP niche.

Most of the marker genes affiliated with *Clostridium* are distributed in two distinct clusters, implying that the contigs belong to at least two different *Clostridium* groups. Interestingly, only one cluster contains the genes of endoglucanases and downstream enzymes for cellulolysis, indicating that this species functions as a helper and the other group does not. Both upstream and downstream cellulose-degrading enzyme genes are also shown in the clusters of *Paludibacter*, verifying that *Clostridium* and *Paludibacter* play a role as helper in the process of cellulose degradation in the consortium. The marker genes affiliated with *Exiguobacterium* and *Tolumonas* lie in the clusters that lack endoglucanase contigs but encode various intracellular-process-related enzymes such as beta-glucosidases, cellobiose phosphorylase, and 6-phospho-beta-glucosidases, strongly supporting our classification of *Exiguobacterium* and *Tolumonas* as beneficiaries.

## Discussion

In the ancient Chinese paper-making procedure, bamboo fibers are softened and soaked in lime water, which would have efficiently extracted the crude hemicellulose (Peng and She, 2014), but retained the crystalline cellulose. A cellulose-rich and alkaline niche has been generated unintentionally in this process, and arguably a microbial community including numerous bacteria and fungi has been evolving intermittently for 7 centuries in the isolated habitat. In this study, we investigated the microbial consortium in such an environment to understand how the microbes interplay *in situ* in cellulose-degradation process using high throughput sequencing. Due to the large size of the fungal genomes, the great diversity of environmental fungi and a small number of fungal reference genomes available, whole genomic analysis of the fungal component in a given microbiome still remains challenging in nowadays. Therefore, considering the complexity of the APMP metagenomic data, we set up a series of stringent criteria in the bioinformatic analyses to focus on bacterial component only and exclude possible errors or bias caused by sequencing depth, wrong assembly, etc. By recruiting 16S rRNA gene reads from the APMP metagenomic sequencing data and assigning the 16S rRNA gene reads taxonomically, we estimated that the reads belonging to the top six abundant genera, including *Cloacibacterium, Paludibacter, Exiguobacterium, Acetivibrio, Tolumonas*, and *Clostridium*, occupy more than 90% of all 16S rRNA gene reads (data not shown), suggesting that the sequencing data may reach more than 100 x depth coverage of the genomes of the top abundant bacteria. The high depth coverage also can be seen from Supplementary Table S5 that the top abundant genera have high number of hits in APMP metagenomic data. To our knowledge, this study firstly reveals that a unique microbial community containing six predominant bacterial genera is present in this isolated habitat and we provide numerous genetic evidences illustrating that these bacteria functionally cooperate with each other in the cellulose-degradation process.

The microbes of this unique niche are presumably novel, because most of the amplicon-based partial 16S rRNA gene sequencing reads were aligned with low identity (less than 90%) against the 16S rRNA gene sequences of the latest RDP Release data^8^ (data not shown). The well-developed software or algorithm, such as QIIME, MetaPhlAn, and so on, may not be suitable for our sequencing data (Kuczynski et al., 2012; Segata et al., 2012). Thus, we developed in-house bioinformatic pipelines for our sequencing data. Firstly, we performed amplicon-based 16S rRNA gene sequencing and developed a bioinformatic pipeline using Naïve Bayesian Classifier to profile the microbial composition on genus-level and phylum-level, respectively, resulting in identifying a unique microbial consortium in the APMP samples compared to other cellulose-rich samples (Demain et al., 2005; Warnecke et al., 2007; Carroll and Somerville, 2009; Zhu et al., 2011; Morris et al., 2012; He et al., 2013; Hummert et al., 2014; Lu and Lu, 2014; Zhou et al., 2014; Dash et al., 2016). Five top abundant genera in the APMP samples were *Cloacibacterium, Paludibacter, Exiguobacterium, Tolumonas*, and *Aeromonas* (Figure 1). Secondly, we performed metagenomic sequencing of the APMP samples to identify the gene content and bacterial taxa, demonstrating that the genera *Cloacibacterium, Paludibacter, Exiguobacterium, Acetivibrio, Tolumonas*, and *Clostridium* are dominant in the APMP samples. Among 672 genera in the database LBPSDB, each of these 6 genera has large number of genes with hits to the genes encoded by the large contigs (≥300 bp) in the APMP metagenomic data (Supplementary Table S5). This result is not completely consistent with that of 16S rRNA gene sequencing data in that none of the abundant taxa in Figure 1 are designated as *Clostridium* or as *Acetivibrio*. This discrepancy can be explained by the limitations of bioinformatic algorithms in identifying novel bacterial taxa from a complex microbial consortium. The key software RDP naïve Bayesian Classifier involved in our bioinformatic pipeline for analyzing 16S rRNA gene sequencing data is trained on the known type strains of 16S rRNA gene sequences (Cole et al., 2005). In contrast, the database LPBSDB that we constructed in this study contains all available 7806 whole genome sequences in NCBI, and these genomes originate from 672 genera. The genera *Clostridium* and *Acetivibrio* that were not shown in Figure 1 have 36 and 2 genomes in the database LPBSDB, respectively. The genes of *Clostridium* and *Acetivibrio* have large numbers of best hits (28742 and 2960, respectively) to the genes encoded by the APMP metagenomic contigs. However, the mean BLAST identities for hits designated as *Clostridium* (around 64%) and *Acetivibrio* (around 61%) are considerably lower than mean identites for the other four dominant genus-level taxa identified by this method (70–86%; Supplementary Table S5), suggesting that those bacteria in APMP samples are likely novel but most closely related to the known strains of the genera *Clostidium* and *Acetivibrio* among the 672 genera in the database of LBPSDB, respectively.

Based on the results of taxonomic assignment and functional annotation of the genes encoded by the APMP contigs (≥300 bp), *Paludibacter* and *Clostridium* species are designated as helpers that encode the entire pathway enzymes to break down cellulose. In contrast, *Cloacibacterium, Exiguobacterium, Acetivibrio*, and *Tolumonas* lack the genes of endoglucanases and are designated as beneficiaries who benefit from the public cellodextrins produced by *Paludibacter* and *Clostridium* species. This finding was further validated by the binning analysis using the software Vizbin (Figure 5). Note that the contigs larger than 1000 bp account for 13% of the contigs larger than 300 bp in the two samples. In the binning analysis, only the contigs larger than 1000 bp were involved, resulting in that a small number of the marker genes are shown in Figure 5. Even so, the binning result significantly verifies that in the microbial consortium, the cellulose-degrading genes are mainly carried by the strains belonging to the six top abundant genera and strongly supports our classification of *Exiguobacterium* and *Tolumonas* as beneficiaries, and *Paludibacter* and *Clostridium* as helpers. However, ambiguous clusters for the contigs of *Acetivibrio* and *Cloacibacterium* shown in Figure 5 indicate the limitations of the binning method (Parker and Hall, 1990; Chauvaux et al., 1995; Dominguez et al., 1995; Schlosser et al., 1999; Shirai et al., 2001; Shaw et al., 2002; Cole et al., 2005; Droge and McHardy, 2012; Kuczynski et al., 2012; Segata et al., 2012; Zheng et al., 2012; Kerepesi et al., 2014; Peng and She, 2014; Laczny et al., 2015).

**FIGURE 6:**
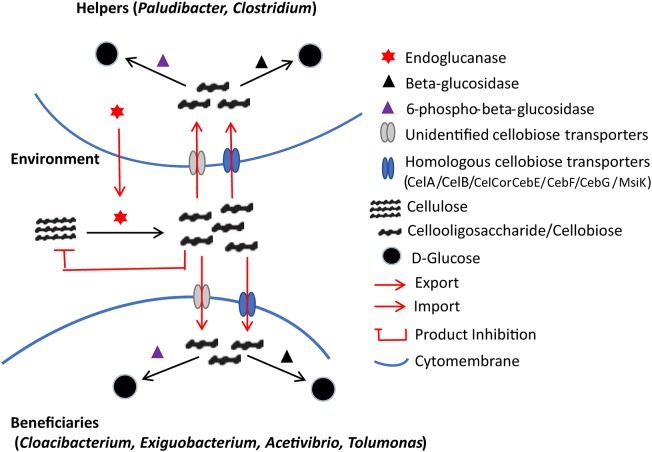
Putative inter-genus interaction network regarding cellulose degradation in the APMP niche. The endoglucanases are produced by helpers and can degrade the macromolecule cellulose into cello-oligosaccharides (cellodextrins). High levels of cellodextrins can inhibit the expression of endoglucanases. Both helpers and beneficiaries encode cellobiose-specific transporters for cellodextrin uptake, followed by intracellular assimilation facilitated by beta-glucosidase, or 6-phospho-beta-glucosidase.

Thus, according to the results of the metagenomic analyses, a putative inter-genera interaction network is shown in Figure 6. The evolution of functional dependency of beneficiaries on helpers generates commensalistic or even mutualistic interactions. Notably, cellobiose products are known to inhibit the cellulolytic process due to the product inhibition effect at moderate to high concentrations (Voutilainen et al., 2008). Therefore, their fast removal by the beneficiaries should alleviate the inhibition (Galazka et al., 2010). The beneficiaries and the helpers likely both get benefits in the APMP consortium, and their interactions can be considered not merely commensalistic but also mutualistic (Schuster et al., 2010).

## Conclusion

Cellulose is a sustainable, cost-effective source of carbon and energy (Carroll and Somerville, 2009). Aside from its use in paper production, cellulose is also under investigation for industrial use, such as conversion into biofuels. The cellulases identified in the APMP consortium may be applied to alkaline pretreatments of cellulosic materials (Wyman et al., 2005), and the new cellulolytic species and the helper/beneficiary strategy revealed by this study may be applied to improve the future design of synthetic microbial consortia, to facilitate engineering of resilient communities, and to achieve high efficiency for industrial cellulose conversion.

## Data Availability

All data are fully available without restriction. All the sequencing data are available from the NCBI SRA database with accession numbers SAMN07807411, SAMN07807412, and SAMN08818942. All data generated or analyzed during this study are included in this published article and its additional files.

## Ethics Statement

All the samples were obtained with consent of the owner of the paper-making plant for scientific research use only and therefore no specific ethical approval is needed.

## Author Contributions

JC, YM, and CL did the experimental design work. JC, GM, and ZW conducted the experiments. GM, JC, YZ, QL, and YM analyzed the data. GM, JC, CL, and YM wrote the manuscript. All authors read and approved the manuscript.

## Conflict of Interest Statement

The authors declare that the research was conducted in the absence of any commercial or financial relationships that could be construed as a potential conflict of interest.

## Footnotes

1 https://www.ncbi.nlm.nih.gov/COG/(September 22, 2016)

2 ftp://ftp.ncbi.nlm.nih.gov/genomes/refseq/(January 09, 2016)

3 http://www.kegg.jp/kegg/tool/annotate_sequence.html

4 http://csbl.bmb.uga.edu/dbCAN

5 www.rcsb.org

6 http://www.tcdb.org/download.php

7 http://amphoranet.pitgroup.org

8 https://rdp.cme.msu.edu

## Abbreviations

APMP: ancient paper-making pulp
BQH: black queen hypothesis
ESS: evolutionary stable strategy
GH: glycoside hydrolase
LBPSDB: latest bacterial protein sequence database
OTU: operational taxonomic unit
PCA: principal component analysis
TCDB: transporter classification database.
