# Supplementary figures and images for: Metagenomic Insights Into a Cellulose-Rich Niche Reveal Microbial Cooperation in Cellulose Degradation
Source: Front Microbiol. 2019 Mar 28;10:618. doi: 10.3389/fmicb.2019.00618 (PMC6447707; doi:10.3389/fmicb.2019.00618)

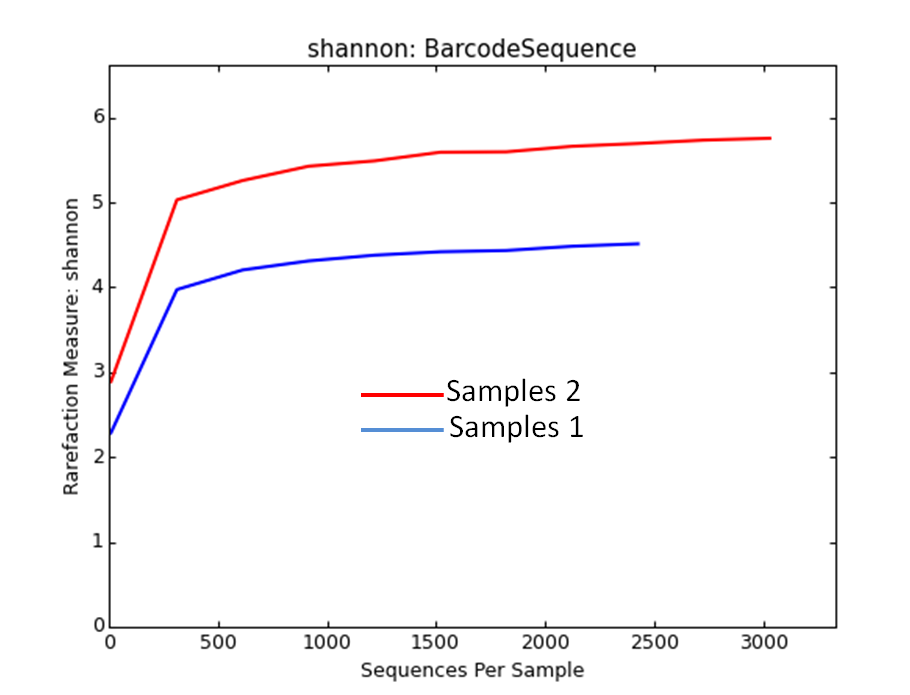

Supplement: Supplementary file 2 [file Image_1.TIF]
